# Early career outcomes of a large four-year MD/ MPH program: Results of a cross sectional survey of three cohorts of graduates

**DOI:** 10.1371/journal.pone.0274721

**Published:** 2023-06-14

**Authors:** Julia Belkowitz, Sabrina Payoute, Gauri Agarwal, Daniel Lichtstein, Roderick King, Shirin Shafazand, Latha Chandran

**Affiliations:** 1 Department of Pediatrics, University of Miami Miller School of Medicine, Miami, Florida, United States of America; 2 Former student Department of Public Health, University of Miami School of Medicine, Miami, Florida, United States of America; 3 Department of Medicine, University of Miami Miller School of Medicine, Miami, Florida, United States of America; 4 University of Maryland Medical System, Baltimore, Maryland, United States of America; 5 Department of Medical Education, University of Miami Miller School of Medicine, Miami, Florida, United States of America; Imam Abdulrahman Bin Faisal University, SAUDI ARABIA

## Abstract

The University of Miami Miller School of Medicine started a four-year MD/ MPH program in 2011 with a mission to graduate public health physician leaders to address the public health needs of the 21^st^ century, with emphasis on three areas: leadership, research, and public health. A prospective cross-sectional survey of early graduates was conducted to understand how they incorporate public health training into their careers. There were two study questions: What are the self-described early career activities of the graduates of the first three cohorts in the areas of leadership, research, and public health and what are the perceptions regarding the influence of the public health training on their careers? In the summer of 2020, a survey was sent to graduates from the classes of 2015, 2016, and 2017. In addition to several multiple-choice questions, the survey included an open-ended question on the impact of public health training in their careers. Inductive content analysis was used to analyze the responses to the open-ended question. Eighty-two of the 141 eligible graduates (63%) completed the survey; 80 of whom had participated or was currently participating in residency training. Forty-nine joined a residency in a primary care field. Many graduates had leadership roles in their early careers, including 35 who were selected as chief residents. Fifty-seven participated in research, most commonly in quality improvement (40), clinical (34) and community based (19). Over one third (30) chose to do work in public health during residency. Themes that emerged regarding the influence of public health training on their careers were: 1) Shifts in perspective, 2) Value of specific skills related to public health, 3) Steppingstone for professional opportunities 4) Focus on health disparities, social determinants, and inadequacies of the healthcare system, 5) Perception as leaders and mentors for peers, and 6) Preparedness for the pandemic. Graduates self-reported involvement in leadership, research, and public health activities as well as a commitment towards addressing some of our most pressing public health needs. Although long-term career outcomes need to be determined over time, graduates currently report considerable benefits of their public health training for their professional outcomes.

## Introduction

It is imperative that the US create a workforce that includes physicians trained in public health who have skills and competencies to address health issues beyond the individual patient to those involving the community and population. In 2007, the Institute of Medicine made a call to enhance public health training among physicians through multiple avenues, including formal degree programs [1]. *Transdisciplinarity* has gained recognition as an approach to solving real world problems that require practical solutions by blurring disciplinary boundaries and integrating the methods of disparate disciplines [2,3]. Integrating public health education into a medical curriculum allows students to gain the skills to address the significant healthcare issues of our time, such as climate change, healthcare inequities, and communicable diseases. Such complex problems require the expertise of both disciplines, each with their unique expertise and tools. Physicians who are trained in both medicine and population and public health sciences play a valuable role addressing these complex, multifaceted issues as they can more easily see potential connections and solutions when the boundaries between these disciplines are dissolved [4].

Many institutions offer dual degree programs to enhance the skill sets of their graduates. Graduates of MD/ MBA programs are less likely to enter primary care specialties and often aspire for leadership positions in healthcare [5–7]. In one study, graduates of combined MD/ PhD programs are no different than their MD only peers in selecting primary care specialties [5]. Other studies report high levels of career engagement in research amongst MD/ PhD program graduates [8]. Currently, 94 of the 157 (60%) US medical schools offer MD/ MPH programs [9]. While no aggregate public data is available regarding the duration of the programs, a special report from the Association of American Medical Colleges (AAMC) indicates that only a portion of the medical schools that offer a MD/ MPH program graduate students within four years [10]. The number of combined programs has been increasing over the past decades, with a total of 822 graduates of combined MD/ MPH programs between 2007–2012 [11] and 1,978 students who graduated between 2014–2019 [10].

In a 2020 study reporting outcomes from a self-reported database of over 18,000 senior medical students, MD/ MPH students self-reported greater levels of leadership, service and research as medical students as compared with MD only peers [5]. Those evaluating career intentions and residency match outcomes comparing MD/ MPH program graduates with MD only graduates found that the former are more likely to match in primary care specialties than the latter [5,11,12]. The definition of primary care varies in the literature; this team of investigators elected to define primary care to include family medicine, internal medicine, pediatrics and obstetrics and gynecology [13–16]. Krousel-Wood et al surveyed graduates who received public health training from one institution 10–20 years post-graduation and found that those with an MPH were more likely to be employed in academia, primary care, and/ or public health and were more likely to conduct research [13]. More detailed understanding of how graduates from MD/ MPH programs focus their careers in public health, community service, research and leadership is needed, and no studies provide insight on how graduates perceive the the influence of such training on their careers.

The University of Miami Miller School of Medicine (UMMSM) curriculum has a mission to “empower medical students to transform lives and inspire them to serve our global community” [17]. Currently, UMMSM has the greatest number of students in the United States (U.S.) graduating with a second degree as compared to all other U.S. medical schools [10]. The four-year MD/ MPH program, founded in 2011, is the longest standing and the largest of its dual degree programs admitting approximately 50 students, a quarter of the student body, each year. Students complete both degrees in four years through seamless integration of the MD and the MPH curricula.

The program is designed to provide students with the academic knowledge and skills, as well as the clinical and public health experience, to improve the health of populations, especially those most vulnerable and underserved. The MD/ MPH program adheres to the competencies required by the Council on Education for Public Health, and students are required to demonstrate competencies in each of the foundational areas. The UMMSM MD/ MPH is offered as a generalist degree with the concentration titled public health physician and is tailored to meet the mission of training its students in “leadership in patient care, research, education, healthcare administration and the community” [18]. Concentration competencies for the MD/ MPH focus on research methodology, economic evaluation, evidence-based medicine concepts, and change management/leadership. The program curriculum includes dedicated coursework on leadership and research methods, and 150 hours of required capstone fieldwork provide the learners with the opportunity to observe and practice leadership skills and experience public health in the community setting. The MD portion of the MD/ MPH curriculum also mandates immersive experiences embedded in local public health department programs in a range of areas from infectious disease to emergency management, among others, and free clinics, public health seminar series featuring active public health physician leaders, and public health clerkship for senior students which centers on care for vulnerable patient populations. As such, the three main tenets of the MD/MPH program are to develop students in the areas of leadership, research, and public health.

Given the described growth of dual public health and medicine programs and relative lack of studies describing whether or not these graduates ultimately incorporate public health training into their careers, this team embarked on a study to better understand the career path of their graduates. There were two study questions: What are the self-described early career activities of the graduates of the classes of 2015–2017 in the areas of leadership, research, and public health and what are the perceptions of graduates regarding the influence of the public health training on their careers?

## Methods

### Study design and participants

All graduates from the first three cohorts of the UMMSM MD/ MPH program were eligible to participate in this cross-sectional survey. All eligible graduates for whom email addresses were available (130/141) were invited to participate in the online survey in July, 2020. Alumni indicated consent to participate in this study by clicking on the survey link. The UMMSM Institutional Review Board approved this study (Protocol #20191104).

### Measures

Demographic information was obtained from data provided by the students during the admissions process. The survey was developed by the study team who agreed upon the selected, adapted, and/ or original questions by identifying those that appropriately addressed outcomes of interest: training and career paths and participation in leadership, research, and public health. Sources for survey items included those available through the American Association of Medical Colleges (AAMC) and other national organizations [19–23]. Demographic data were collected and included gender identity, marital status, number of dependents, and specialty of residency training as per the AAMC Graduation Questionnaire [19]. Race and ethnicity were defined according to the federal government definitions [20]. Types of residency training program was defined based on the AAMC Program Survey [21]. Graduates were also asked to identify age, graduation year and status of training in residency and fellowship (as applicable).

Questions about current workplace setting were adapted from AAMC and American Academy of Pediatrics surveys [19,22,23]. Items regarding involvement in leadership, research and public health were novel and included yes/ no or multiple responses with the opportunity for free text when graduates affirmed participation in a specific area (Table 1). The single open-ended question was “Please describe the impact of your public health education on your career.” The full survey is available upon request. Overall, the survey contained 29 optional items, sixteen of which included the opportunity for free text or additional options if the participant affirmed. Survey logic also excluded questions when not applicable based upon the current training status or response. The survey participation time was estimated to be 10–15 minutes.

**Table 1:** Survey items for leadership, research, and public health.

**Leadership**
Were/ are you selected to be a chief resident?
Other than chief resident, did you/ do you hold other leadership positions as a resident?
Do you hold/ have you held leadership positions in your specialty organization?
Do you hold/ have you held other state or national leadership positions?
Have you received any awards?
**Research**
Do you participate in research activities?
Have you applied for external funding (federal, foundation grants)?
**Public Health**
Were you required to do any public health work during your residency training?
Outside of what was required, did you elect to do any public health work during your residency training?
What is the best description of your current work status?
What is the best description of your current place of practice?
Please indicate the setting in which you currently work.
Do you work in a primarily underserved area?
Please describe the impact of your public health education on your career.

### Data analysis

Survey data was collected using Redcap v11.2.2 and de-identified for analysis. Quantitative data was reported using tabular counts and descriptive statistics. A Chi-Square test for association was used to determine if there was any association between demographics (gender, race/ethnicity, academic year of graduation) of survey respondents compared to overall MD/ MPH class of 2015–2017. Data were analyzed in R-Studio. A p-value of 0.05 was considered statistically significant in these analyses.

Brief free text responses were independently summarized into categories by three investigators (JB, SP, GA) and the group discussed to achieve consensus when there was initial disagreement about the classification.

Qualitative analysis was done manually for the one open-ended question using the method described by Elo and Kyngas for inductive content analysis [24]. In the preparation phase, it was determined that the unit of analysis would be each phrase in an individual’s response. Two investigators (JB and SP) independently reviewed the responses for the process of open coding and together established sub-categories to create a codebook summarizing the definition of the sub-categories and explanation for how to apply the codes during the analysis. In order to improve credibility through investigator triangulation, three investigators (JB, SP and GA) then independently reviewed the free responses and applied the sub-categories to the collected data [25]. This team then met together regularly for the abstraction process, consolidating the responses into generic categories and then broader main categories that illustrated the perspectives of the participants about their experiences. When there was initial disagreement, the three team members discussed until consensus was achieved.

## Results

### Demographics characteristics

Of the 141 eligible participants, contact information was available for 130 alumni who were then invited to participate. This included 47/48 from the class of 2015 (98%), 45/48 from the class of 2016 (94%), and 38/46 (83%) from the class of 2017. A total of 82 alumni from the three cohorts responded to the questionnaire, with 31(66%), 29 (64%), and 22 (58%) responding, respectively, from the classes of 2015, 2016, and 2017, resulting in an overall response rate of 63%. Demographic characteristics at enrollment in the survey are summarized in Table 2. There were no statistically significant differences between survey respondents and overall MD/ MPH program graduates of the demographic factors available for analysis: gender, race and ethnicity, and academic year of graduation. Females represented 54% of survey respondents (44). Among survey respondents, 56% (46) self-identified as white, 5% (4) Black or African American, 13% (11) Asian, 9% (7) Hispanic, or Latino, or of Spanish origin, 4% multiracial (3), and 11(13%) other/ not specified race. Seventy three percent (60) of respondents claimed zero dependents. Regarding marital status, 55% (45) of survey respondents were married. The average respondent age was 31.6 years (range 28–36 years).

**Table 2:** Demographic characteristics of survey respondents versus the overall MD/ MPH cohort.

	Total (n = 82) Respondents	Overall Program (n = 141)	P value
	Number (percent)	Number (percent)	
**Gender**^**1**^ Female Male	44 (53.7) 38 (46.3)	86 (61.0) 55 (39.0)	0.054
**Race and Ethnicity**^**1**^ Asian Black or African American Hispanic, Latino, or of Spanish origin Multiple Race and Ethnicity White Other or Nonspecified	11 (13.4) 4 (4.9) 7 (8.5) 3 (3.7) 46 (56.1) 11 (13.4)	26 (18.4) 8 5.7) 10 (7.1) 4 (2.8) 75 (53.2) 16 (11.3)	0.4624
**Academic Year of Graduation**^**1**^ 2015 2016 2017	31 (37.8) 29 (35.4) 22 (26.8)	48 (34.0) 46 (32.6) 47 (33.3)	0.1531
**No. of Dependents (not including spouse/partner), at time of survey** 0 1 > 1	60 (73.2) 14 (17.1) 8 (9.8)	Not available	N/A
**Marital Status at time of survey** Single (never legally married) Legally married Other	32 (39.0) 45 (54.9) 5 (6.1)	Not available	N/A
**Age at time of survey, years** Mean (SD): 31.6 (1.8) Range: (28–36)		Not available	N/A

^1^Demographic data collected at the time of admission.

### Residency and fellowship training

**Fig 1:**
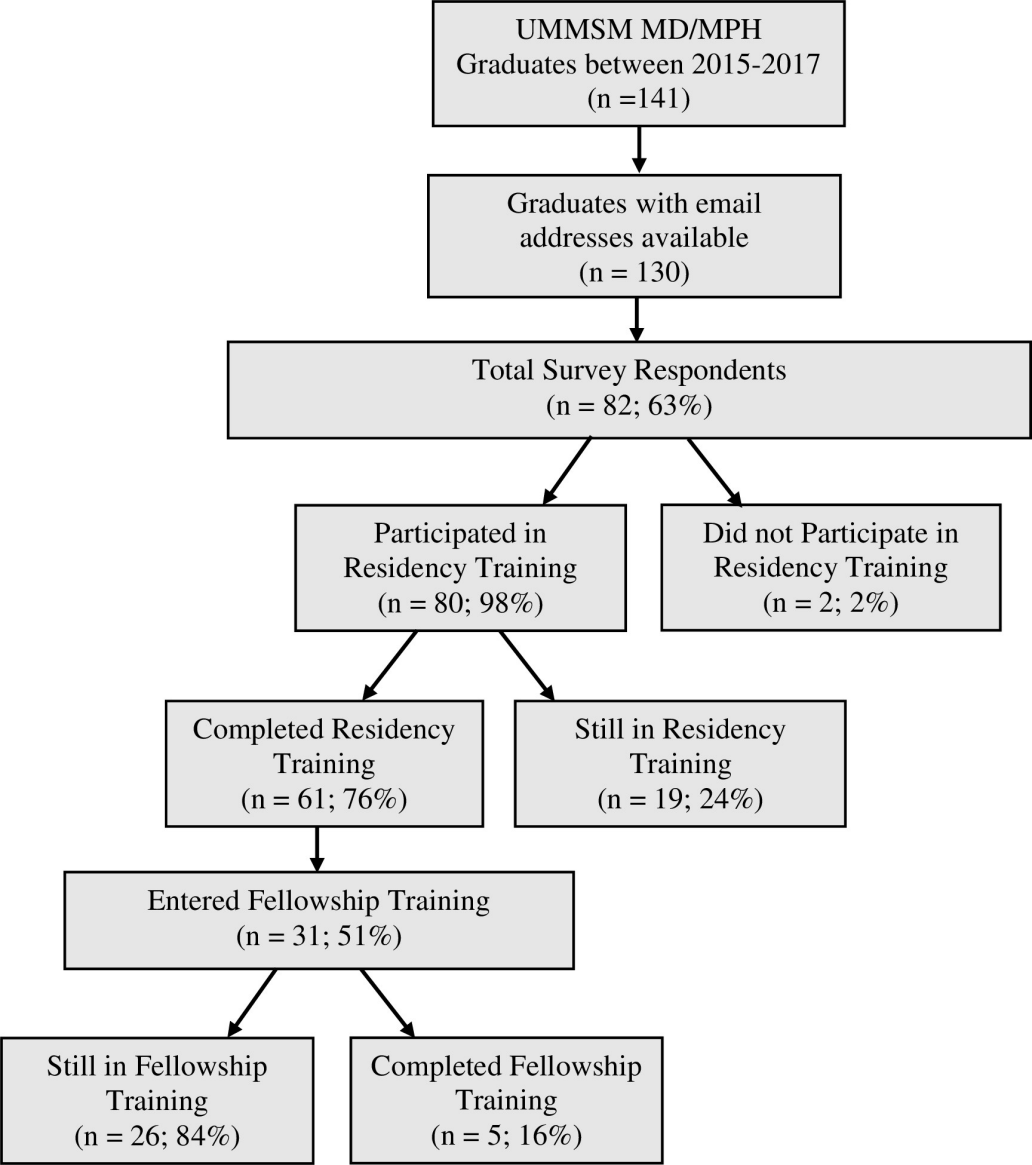
UMMSM MD/ MPH alumni training status.

**Fig 2:**
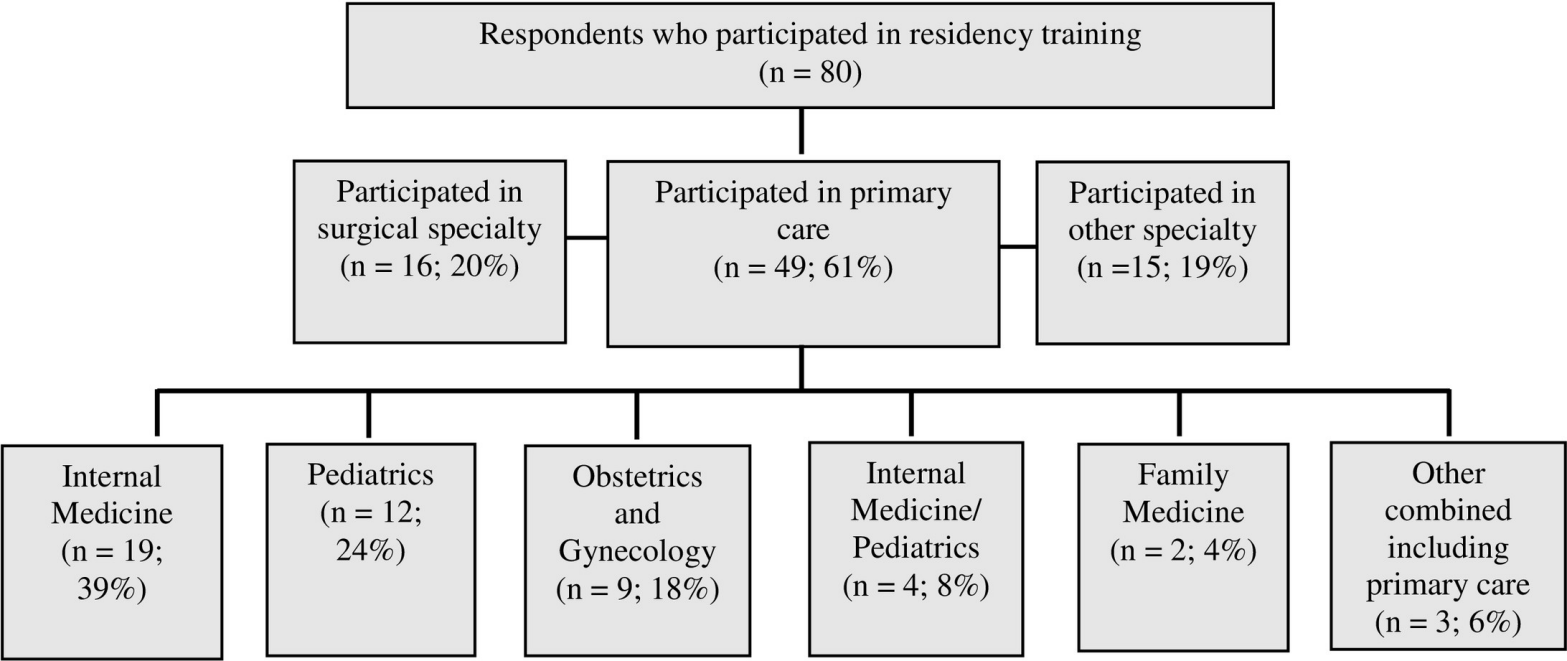
UMMSM MD/ MPH alumni specialty choice.

Of the total of 82 respondents, 80 (98%) who participated in residency or fellowship training are included in the analysis. Of these, 35 participants had completed their graduate medical education. Details regarding status of training and specialty choice are depicted in Figs 1 and 2. Eighteen (23%) participated in a specialized track for residency training. Graduates in a specialized residency track reported to have participated in pathways such as Advocacy/ Community Health (5), Primary Care (4), Global Health (3), and others.

### Leadership, research, and public health

Many MD/ MPH program graduates reported that they held leadership positions either currently or in the past. Leadership characteristics are summarized in Table 3. In this table overall responses are summarized in one column, and the responses for those who have completed their Graduate Medical Education (GME) training (residency and fellowship training, as applicable) in another. Thirty-five (44%) were selected as chief residents. Thirty-eight (48%) listed other leadership activities held as a resident, 12 (15%) held leadership positions in their specialty specific organizations, and ten (12%) have held other state/national leadership positions. Categories of participation included leadership roles within their residency program or representing their residency program such as curriculum committee, GME representative (n = 26); leadership within their specialty specific organization (ex. national delegate or committee chair), (n = 18); leadership in research, patient safety, and/ or quality improvement (n = 11); leadership regarding specific professional domains in the areas of diversity, wellness, and interprofessional collaboration (e.g. Diversity in Equity and Inclusion Committee), (n = 10); leadership in community-based activities (e.g.. Community Health Committee) (n = 5); and leadership related to clinical algorithms or skills development (eg. skills lab instructor) (n = 4). Over half of those who had undergone residency training (n = 42, 53%) reported receiving a professional award. Categories included awards highlighting their excellence as a resident/clinical skills from either the residency program or the specialty society (n = 33); awards for research/ scholarship or patient safety/ quality improvement (n = 21); awards for teaching/ humanism/ AOA (n = 16); and other awards (n = 12).

**Table 3:** Leadership, research, and public health post medical school graduation.

Domain	Sample participation types	Participation Overall (n = 80)	Participation among those who have completed GME training (n = 35)
**Leadership**		N (percent*)	N (percent*)
	Selected as chief resident	35 (43.8)	15 (42.9)
	Held other leadership activities as a resident	38 (47.5)	16 (45.7)
	Held leadership positions in their specialty specific organization	12 (15.0)	5 (14.3)
	Held other state/national leadership positions	10 (12.5)	5 (14.3)
	Received an Award	42 (52.5)	19 (54.3)
**Research**			
	Participated in Research	57 (71.3)	19 (54.3)
	**Research Type: Quality improvement projects Investigator initiated clinical research Community-based research Translational research Basic science research Industry sponsored trials	40 (50.0) 34 (42.5) 19 (23.8) 14 (17.5) 6 (7.5) 3 (3.8)	14 (40.0) 10 (28.9) 7 (20.0) 2 (5.7) 2 (5.7) 1 (2.9)
	Applied for external grant funding	11 (13.8)	5 (14.3)
	**Research grant funding support: Federal (DOD, NIH) Foundation grants Academic Society grants Industry sponsored educational grants	2 (2.5) 7 (8.8) 6 (7.5) 1 (1.3)	2 (5.7) 2 (5.7) 4 (11.4) 0 (0.0)
**Public Health**			
	Required to do public health work during residency training Yes No	8 (10.0) 72 (90.0)	3 (8.6) 32 (91.4)
	Chose to do public health work during residency training outside of what was required	30 (37.5)	12 (34.3)
	Description of current work status Direct healthcare delivery only Both medicine and public health/ administration	69 (86.3) 11 (13.8)	29 (82.9) 6 (17.1)
	Description of current place of practice Military Academic position Solo or group practice HMO (staff model) Non-government hospital Government hospital Non-profit community health centers *** Other	4 (5.0) 35 (43.8) 9 (11.3) 2 (2.5) 9 (11.3) 2 (2.5) 1 (1.3) 18 (22.5)	4 (11.4) 11 (31.4) 8 (22.9) 1 (2.9) 7 (20.0) 0 (0.0) 1 (2.9) 3 (8.6)
	****Setting of current work Large City (500,000 or more) Suburb of a large city Moderate City (50,000–500,000) Small City (10,000–50,0000) Rural/ Unincorporated Area	39 (48.8) 4 (5.0) 16 (20.0) 1 (1.3) 1 (1.3)	18 (51.4) 4 (11.4) 12 (34.3) 0 (0.0) 1 (2.9)
	****Work in a primarily underserved area	31 (51.6)	22 (62.9)

*Percentages may not add up to 100% due to rounding.

**Note: Percentage do not add up to 100 due to participants being able to select more than one type.

***Other: Includes free-standing ambulatory care, surgical, or emergency care centers.

****Only participants who completed residency training (n = 61).

Fifty-seven (71%) MD/ MPH program alumni reported participation in research. Research characteristics are summarized in Table 3. Forty (50%) participated in quality improvement projects, 34 (43%), participated in investigator initiated clinical research, 19 (24%) in community-based research, 14 (18%) in translational research, 6 (8%) in basic science research, 3 (4%) in industry sponsored trials. Among the 57 that participated in research, 11 (14%) successfully received research grant support from sources including federal agencies (2, 3%), foundation (7, 9%), academic society (6, 8%), and industry sponsored grant funding (1, 1%).

Results regarding the graduates’ involvement in public health are summarized in Table 3. Over a third of the respondents, 30 (38%) voluntarily chose to participate in public health work during residency, outside of what was required of them. Overall, categories of the public health related work were included Advocacy/ leadership (n = 14), Research/ patient safety/ quality improvement (n = 12), community based (n = 10), global health (n = 8), clinically related (n = 6), teaching (n = 5).

**Fig 3:**
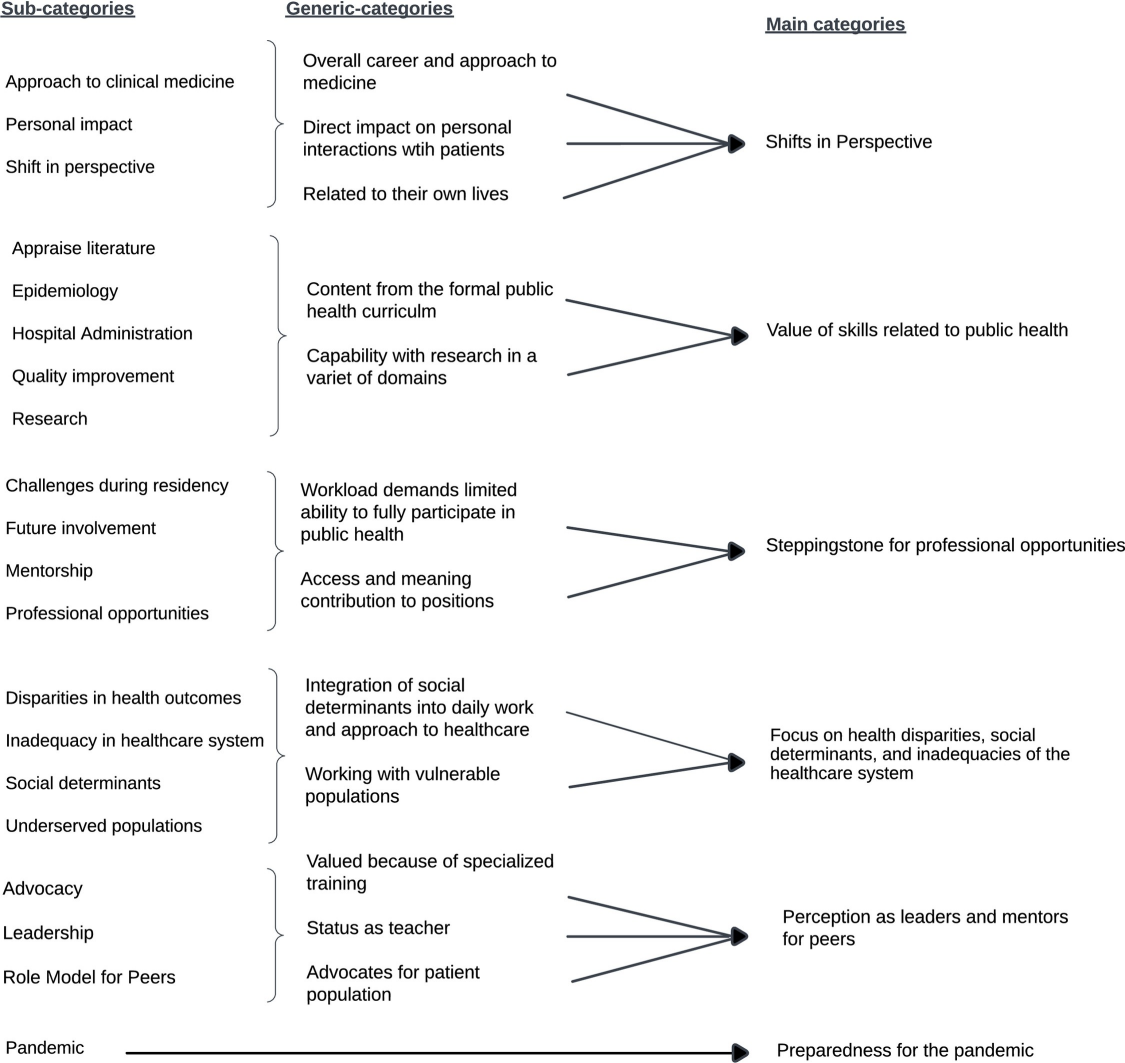
Summary of the inductive content analysis process.

There were 69 responses to the optional open-ended question “describe the impact of your public health education on your career.” Response length ranged from 8–202 words, with a median of 32 words. All responses were in included in the analysis. Fig 3 provides a summary of the components of the inductive content analysis. For example, the sub-categories “approach to clinical medicine,” “personal impact,” and “shift in perspective” consolidated to generic-categories of “overall career and approach to medicine,” “direct impact on personal interactions with patients,” and “related to their own lives” that were grouped into the main category of “shifts in perspective.” Content analysis of the responses revealed six broad themes: 1) Shifts in perspective, 2) Value of specific skills related to public health, 3) Steppingstone for professional opportunities 4) Focus on health disparities, social determinants and inadequacies of the healthcare system, 5) Perception as leaders and mentors for peers, and 6) Preparedness for the pandemic.

#### Shifts in perspective

Alumni report that participation in the MD/ MPH program had a significant impact on shifting their perspective regarding both their overall career and approach to clinical medicine. Some examples include:

*“My public health education has shaped the way I practice medicine in many ways*. *Every time I see a patient I try and get to the root cause of their problem(s)*.*”*

*“Public health has formed the way I view medicine*. *My perspective is different because of my public health training*. *I view things from a systemic perspective in addition to an individual patient perspective and try to be cognizant of systemic issues which prevent adequate health in the community in which I practice*.*”*

Others share a shift in a more direct impact on their personal interactions with patients or in their own lives:

*“Changes the way I treat patients*. *Helps me connect on a deeper level with people from all backgrounds*.*”*

*“My public health education taught me to better understand why underserved populations have worse health outcomes*, *which has inspired me to actively work to eliminate this health outcome gap by working on implicit personal level and system level biases in the way I practice medicine and live my life*.*”*

#### Value of specific skills related to public health

Content from the formal public health curriculum such as epidemiology, quality improvement, critical appraisal of literature was identified by graduates as important for their current day to day work as exemplified here:

*“My [public health] education has given me the ability to easily critically appraise medical literature and understand the meaning of biostatistics*.*”*

*“The biggest impact I have found is how my training has helped me interact with hospital administration to improve patient care in both the inpatient and outpatient center*.*”*

Graduates especially highlighted the impact of their training on their participation in and capability with research in a variety of types of domains including community based, clinical, and global health projects:

*“The coursework of the MPH degree has proven useful in clinical research*, *project proposals*, *grant applications*, *and publication in peer-reviewed journals*.*”*

*“I gained substantial research and statistical training through my public health education that I continue to apply to my current research in high-risk women and infectious disease*.*”*

*“My public health background has encouraged me to push for or find a solution to increase minority/ underserved population inclusion in cancer clinical trials*. *I initiated a retrospective study based on rising costs of [disease management] to find targeted areas to help with this issue*.*”*

#### Steppingstone for professional opportunities

Alumni commonly expressed that workload demands limited their opportunity to fully participate in public health while in training, they still plan to incorporate public health into their future careers,

*“While much of residency has been taken up with clinical duties*, *I’m still hoping to pursue a research focus in healthcare disparities as an attending*.*”*

Others lauded how their public health credentials enabled them access and allowed them to meaningfully contribute to positions they may not otherwise have had, both during training and for early career opportunities.

*“My training also made me better suited to function as a leader and community liaison for the [community-based program]*.*”*

*“My public health education has been incredibly important for my career*. *It has allowed me to understand public health and to pursue [professional opportunity] which has been an amazing experience*.*”*

#### Focus on health disparities, social determinants and inadequacies of the healthcare system

Alumni frequently emphasized and prioritized these areas when describing their day-to-day work and approach to healthcare:

*“It created a strong foundation of the importance of population health and understanding how social determinants have a huge impact on individuals health and further more on communities*.*”*

*“Aspects of my clinical research and quality improvement projects and seek to improve healthcare systems and delivery of care within our [clinic]*. *I feel strongly that it is my public health background that has provided the skills and knowledge to carry all of this out…”*

*“Exposed me to working collaboratively and leading groups around population- specific priorities and opportunities for improvement in healthcare delivery and health promotion*.*”*

Many graduates also expressed that their experiences in medical school propelled them towards working with vulnerable populations,

*“I work at [county’s] public safety net hospital and am committed to a career in service to underserved populations*.*”*

#### Perception as leaders and mentors for peers

Many alumni believe they were viewed as leaders in their residency/ work because of their specialized training.

“Set me apart from my peers as being uniquely qualified in healthcare systems-thinking and related leadership roles”

*“My co-residents often come to me for help with their quality improvement projects and questions regarding to interpreting the medical literature because they know that I have had public health and research training*.*”*

Teaching was identified as an important aspect of this status,

*“…Also*, *as a public health physician I try and educate my colleagues and patients on the importance of public health*.*”*

Graduates also explained that they viewed themselves as advocates for their patient population:

*“…it pushed me to strengthen my skills in advocating for my patient and finding the right resources for them*.*”*

#### Preparedness for the pandemic

MD/ MPH program alumni shared that they felt especially equipped to be working in the healthcare system as the COVID-19 pandemic emerged. As explained by one graduate:

*“It has also helped me to understand the COVID pandemic at the epidemiologic and socio-political levels*, *even from very early on*.*”*

## Discussion

This study examines early career outcomes and qualitative comments regarding their experience from a large cohort of MD/ MPH students. These study results indicate that the graduates of the UMMSM dual degree MD/ MPH program are utilizing their public health education and continue to remain committed to leadership, research, and public health several years after graduation. The graduates’ choices of post-graduate programs (residencies) indicated that over 50% of them chose primary care specialties or combined primary programs that included primary care. The tendency towards MD/ MPH graduates to more heavily choose primary care residencies is consistent with graduate intentions and match outcomes at other institutions [5,11,12]. In a long-term study which followed graduates from one institution’s MD/ MPH program for 10–20 years post-graduation, members of their MD/ MPH cohort were more likely to practice general primary care [13]. It remains to be seen if this institution’s graduates will indeed follow a similar path as, 21 (43%), of these graduates who initially entered primary care related residencies ultimately participated in fellowship training. Future studies investigating long-term career pathways will be required to better understand their ultimate career trajectories.

Nonetheless, the mission of the dual degree program at this institution is not rooted in training primary care physicians but rather, public health physician leaders, regardless of specialty choice. Christensten and colleagues described residency characteristics comparing MD/ MPH students with MD only graduates and found that MD/ MPH students had significantly more participation in Gold Humanism Honor Society (peer selected society that recognizes compassionate care in medicine) membership, research related experiences, leadership experiences, and volunteer experiences during medical school [5,26]. The results of the current study indicate that they are indeed beginning to build a foundation to become public health leaders. Forty four percent of the program’s alumni were selected as chief residents, and 48% held some other leadership role as a resident outside of serving as chief. Narrative comments also supported their trajectory towards becoming public health leaders through their self- identified positions in their residency program as role models and teachers. Graduates of other MD/ MPH programs are more likely to describe intentions of working in academic medicine, healthcare administration and/or state and federal administration [11]. However, MD/ MPH programs are not the only dual degree programs who produce leaders. For example, other studies of MD/ MBA students found that they commonly aspired for organizational leadership in healthcare, though long term outcomes were not assessed [6,7]. Further study will be required to determine how much the MPH impacts leadership outcomes, especially as compared with other dual degree programs.

In terms of research, 71% percent of the graduates participated in research since graduation, with 50% involved in quality improvement related work and 24% in community-based research. Not surprisingly, MD/ PhD graduates are reported to engage in even greater levels of research after medical school graduation and GME training [8]. Yet, only a small number of MD/ MBA graduates report considering a path as a physician scientist [6].

While other dual degree programs may be posed to training leaders and researchers, an important question is whether public health training in medical schools will lead to an expansion of the workforce of public health professionals. Emerging findings from this study and others seem to support that it will. More than one third (38%) of the graduates voluntarily elected to participate in public health endeavors during residency above and beyond what was required in their respective curricula, despite the rigorous demands of their schedules and residency responsibilities. Perhaps most meaningful in evaluating the role public health education (MPH) played in the careers of the respondents were the themes that emerged from the rich narrative comments in response to the open-ended question. Not surprisingly, students appreciated the specific public health skills such as appraisal of the literature and research. Others have surveyed medical student participants of their MPH training programs and similarly reported improved skills in critical appraisal of the literature and research skills, as well as a commitment towards underserved populations, policy and global health [14,27]. A national study of a cohort of 822 MD/ MPH graduates found that MD/ MPH program graduates were more likely to report altruism as a factor in their career choice than MD only graduates [11]. These findings are also highlighted in the comments from our study participants in themes that emerged regarding dedication towards addressing social determinants and service towards vulnerable populations, among others. There are many reasons why the integrated dual degree curriculum could impact the commitment towards public health work, including that the students themselves who matriculate inherently are more interested in such work. Additional reasons could be the previously described curriculum that is tailored specifically for public health physicians. The transdisciplinary and integrated nature of learning both public health and medicine at the same time may allow for an enhanced relevance of both curricula on the learner.

The research question for this study centered simply on describing early career outcomes for a cohort of graduates from one institution’s program. There was no comparison group either within or outside of the single institution. Inferences cannot be made regarding superiority of one curricular model over another nor over other dual degree programs at this and other institutions. These questions leave room for further study, including whether the integration in the curriculum in a four-year model, rather than a stand-alone fifth year, improves the leadership, research and public health career outcomes in MD/ MPH students. However, it is clear that a four year integrated program, rather than a five year one, reduces both the time and financial burden on students. It is possible that this alone may garner more interest from potential applicants and could potentially benefit our public health physician workforce both in terms of volume and reduction of time prior to job entry. This study team intends to follow this and future cohorts over time to better describe the ultimate career outcomes of the institution’s graduates.

Limitations of this study include that the data was self-reported using a survey that had not been formally validated. There is always a potential for self-selection bias given the methodology. Although it allowed for a “no impact” response, asking respondents about the impact their public health training on their careers could potentially create a confirmation bias. As previously noted, in this first survey, there was no comparison group; future research should include students from the institution’s MD and other dual degree pathways. The most common category of leadership in which these participants participated is quality improvement. Given that some residencies may require such work, it is difficult to determine if these results indicate an innate interest in research overall. It is also difficult to make predictions regarding the long-term involvement of study participants in leadership, research, and public health. The investigators plan to follow these and future cohorts of graduates to determine the level of their continued engagement in these areas.

This study suggests that graduates from a four-year integrated MD and MPH curriculum remain involved as public health physician leaders. Though the full impact of this training cannot be measured for many years to come, it is encouraging to see the self-described early career activities of MD/ MPH trained physicians. The most recent pandemic clearly laid bare the need for excellence in public health and its true integration with medical care. Developing graduates with transdisciplinary skills who are skilled in medical care and public health without the commitment of additional training time can be quite helpful in dealing with the health care work force crisis. Such programs can enhance the workforce with skilled health care workers who have the potential to respond appropriately to future infectious and environmental emergencies that may emerge. It is the responsibility of the medical education community to continue to expand similar training programs that integrate the public health work force needs with the idealism and altruism that many students bring to their medical training.
